# Application of the distally based sural neurocutaneous flaps in the management of foot and ankle defects in patients with diabetic foot

**DOI:** 10.3389/fendo.2022.1009714

**Published:** 2022-09-23

**Authors:** Jiezhi Dai, Yu Zhou, Shasha Mei, Hua Chen

**Affiliations:** ^1^ Department of Orthopedic Surgery, Shanghai Jiao Tong University Affiliated Sixth People’s Hospital, Shanghai, China; ^2^ Department of Orthopedic Surgery, Civil Aviation Hospital of Shanghai, Shanghai, China; ^3^ Department of Anesthesiology, Shanghai Jiao Tong University Affiliated Sixth People’s Hospital, Shanghai, China

**Keywords:** diabetic foot, diabetic wound defect, distally based sural flap, wound healing, foot and ankle reconstruction

## Abstract

**Background:**

We report our experience on the use of a distally based sural flap for soft tissue reconstruction of foot and ankle defects in patients with diabetic foot.

**Methods:**

The actual study is a retrospective, open, non-controlled, and clinical study of 25 patients treated with diabetic foot on whom reconstruction with distally based sural neurocutaneous flaps was performed from May 2019 to December 2021.

**Results:**

The mean age was 64.9 years, and there were 15 male and 10 female patients. The mean follow-up was 9.8 months, which ranged from 6 to 12 months. The size of the flaps ranged from 6 × 5 to 15 × 9 cm^2^. Twenty-two of the 25 flaps survived intact with sufficient blood supply. Two cases had a small superficial necrosis, which was resolved after a change of daily dressing and was heeled eventually. In one case, partial necrosis was observed that was managed with minor revision and the use of split-thickness skin graft.

**Conclusions:**

The distally based sural flap is considered to be useful for reconstruction of foot and ankle defects in patients with diabetic foot.

## Introduction

Diabetic foot is one of the most significant and devastating complications of diabetes (1). Impaired wound healing in patients with diabetes can lead to infections, chronic ulcers with a recurrence rate of 66%, and even lower extremity amputation, which significantly affects the patients’ quality of life (2).

In patients with diabetic foot, reconstruction of soft tissue in the distal lower extremities is a significant challenge as a result of peripheral neuropathy and diabetic microangiopathy (3). After primary debridement, it is difficult to obtain a primary closure for the subsequent defect in foot and ankle. Skin grafts are usually unacceptable when bones or tendons are exposed. Local flaps are not always reliable because of the presence of varying severity of peripheral arterial disease (4). Free flap transfer allows for more options and is widely used in recent years. Despite the high success rates of free flaps even in patients with diabetes, pronounced microsurgical skills and proper case selection are required (5).

In 1992, Masquelet et al. first introduced the concept of neurocutaneous island flaps supplied by the vascular axis of the sensitive superficial nerves in the leg (6). Since then, the distally based sural flap has received more attention and eventually became a mainstay in the reconstruction in the lower leg, ankle, and heel (7). Many studies have been reported the versatility of the distally based sural flap for soft tissue coverage originated by traumatic or infectious events (8). However, there are few reports on the use of these flaps as a reconstructive option in patients with diabetic wound. In this study, we presented our experience on the use of distally based sural neurocutaneous flaps for coverage of foot and ankle defects in patients with diabetic foot ulceration.

## Material and methods

This study is a retrospective, open, non-controlled, and clinical study of patients with diabetic foot on whom reconstruction with distally based sural neurocutaneous flaps was performed from May 2019 to December 2021 at the Department of Orthopedic Surgery, Sixth People’s Hospital affiliated to Shanghai Jiao Tong University, Shanghai, China. This retrospective study was approved by our institutional review board.

Patients who were diagnosed with type 2 diabetes based on the diagnostic criteria recommended by the American-Diabetes-Association (ADA) in 2010 were included (9). DFU is defined as “ulceration of the foot (distally from the ankle and including the ankle) associated with neuropathy and different grades of ischemia and infection”, according to the World Health Organization (10). All of the included patients had preservation of at least one major artery (anterior tibial artery, posterior tibial artery, and peroneal artery). We excluded chronic wound caused by pressure ulcer, vasculitis, pyoderma gangrenosum, and diseases that cause ischemia (11).

In this study, we recorded patients’ age, sex, duration of diabetes, location of defect, size of defect, size of flap, outcomes, postoperative complications, and follow-up. All surgery was performed by one surgeon (CH). Appropriate medical treatment included blood glucose regulation, perfusion improvement by prostaglandins or antiplatelet drugs, appropriate antibiotics administration, and routine sterile dressing change.

### Surgical technique

In debridement, we removed and debrided non-viable infected soft tissues and bones. The edges of debridement were achieved until the soft tissues and bones presented to be generally healthy.

After debridement, the flap was designed with respect to the defect. The patient was placed in a prone position, with the application of a tourniquet. A line of incision was traced over the presumed course of the sural nerve and the lesser saphenous vein. The pivot point of the pedicle was preoperatively marked 5 cm proximal to the tip of the lateral malleolus. The flap was harvested including the medial sural nerve, the lesser saphenous vein, and the deep fascia. The incision was made along the superior border of the flap, and the terminal perforator was easily found at the fascial plexus of the flap and was carefully dissected. The flap was then harvested in the subfascial plane and rotated from 90° to 180° to cover the recipient site and inset without tension. The donor site defect was reconstructed using a skin graft or finally closed primarily.

## Results

A total of 25 patients with diabetic wound were included in this study. There were 15 male and 10 female patients. The mean age of the patients was 64.9 years. The detailed clinical characteristics of the patients are presented in **Table 1**. The mean follow-up was 9.8 months, which ranged from 6 to 12 months.

**Table 1:** Summary of the patients receiving distally based sural neurocutaneous flaps for foot and ankle reconstruction.

Case	Gender	Age	BMI (kg/m^2^)	Duration of diabetes(years)	Ulcer location	Smoking (yes/no)	HbA1c(pre-op)	Renal function	Complicated with osteomyelitis (yes/no)	Wagner classification(grade)	Flap size (cm^2^)	Wound size (cm^2^)	Outcome	Follow-up (months)
1	M	64	22.41	5	Plantar midfoot	No	10.2	Normal	No	2	15*6	12*4	Survived completely	12
2	M	67	21.53	13	Plantar hindfoot	Yes	9.8	CKD II	No	2	10*5.5	8*4	Survived completely	9
3	M	59	22.12	6	Fourth and fifth toes and lateral forefoot	Yes	15.8	Normal	Yes	3	12*6	10*4	Survived completely	9
4	M	78	21.76	22	Plantar hindfoot	No	13.7	CKD II	No	3	14*7.5	11*6	Superficial necrosis	9
5	M	55	25.25	5	Dorsal midfoot	Yes	12.4	Normal	No	3	9*8	7*7	Survived completely	9
6	M	62	23.34	9	Fifth toe and lateral forefoot	Yes	11.7	CKD I	Yes	3	14*5	12*3	Survived completely	9
7	M	65	22.38	12	Plantar midfoot	Yes	13.2	CKD I	No	3	12*7	11*5	Survived completely	9
8	M	73	22.11	18	Plantar hindfoot	Yes	11.9	CKD II	No	3	10*8	8*6	Survived completely	9
9	M	66	23.34	13	Lateral ankle	Yes	16.6	CKD II	Yes	4	14*8	11*6	Survived completely	12
10	M	69	23.98	11	Plantar hindfoot	Yes	14.1	Normal	No	3	12*7	9*5	Survived completely	9
11	M	72	22.56	20	Behind the Achilles tendon	No	8.9	Normal	No	2	6*6	4*3	Survived completely	6
12	M	61	22.34	10	Lateral ankle	No	9.2	CKD I	No	2	5*6	5*5	Survived completely	6
13	M	58	24.26	4	Lateral ankle	No	12.7	Normal	No	3	9*7.5	6*5	Survived completely	9
14	M	63	22.75	4	Dorsal midfoot	Yes	10.3	Normal	No	2	8*6	5*5	Survived completely	6
15	M	71	21.25	9	Plantar hindfoot	Yes	9.6	CKD I	No	2	11*9	10*7	Survived completely	12
16	M	68	20.77	5	Plantar hindfoot	Yes	12.6	CKD I	No	3	10*6	9*4	Survived completely	12
17	F	65	21.54	8	Fifth toe and lateral foot	No	13.1	Normal	Yes	3	15*9	13*6	Partial necrosis	12
18	F	63	22.36	11	Plantar hindfoot	No	15.8	CKD II	Yes	4	14*9	12*6	Superficial necrosis	12
19	F	67	21.83	15	Heel	No	12.1	CKD I	No	3	12*8	10*5	Survived completely	9
20	F	62	22.42	6	Medial ankle	No	11.9	Normal	No	3	11*6	9*4	Survived completely	12
21	F	59	21.78	4	Dorsal midfoot	No	8.7	Normal	No	2	10*7	7*5	Survived completely	9
22	F	69	22.13	12	Fifth toe and lateral plantar foot	No	11.4	CKD I	Yes	3	12*8.5	10*5	Survived completely	12
23	F	61	21.35	3	Heel	No	10.2	Normal	No	2	8*8	6*6	Survived completely	12
24	F	63	20.67	5	Second and third toes and dorsal forefoot	No	11.2	Normal	Yes	3	11*6	9*5	Survived completely	12
25	F	62	21.81	7	Plantar hindfoot	No	9.3	Normal	No	3	10*5	7*3	Survived completely	9

**Figure 1:**
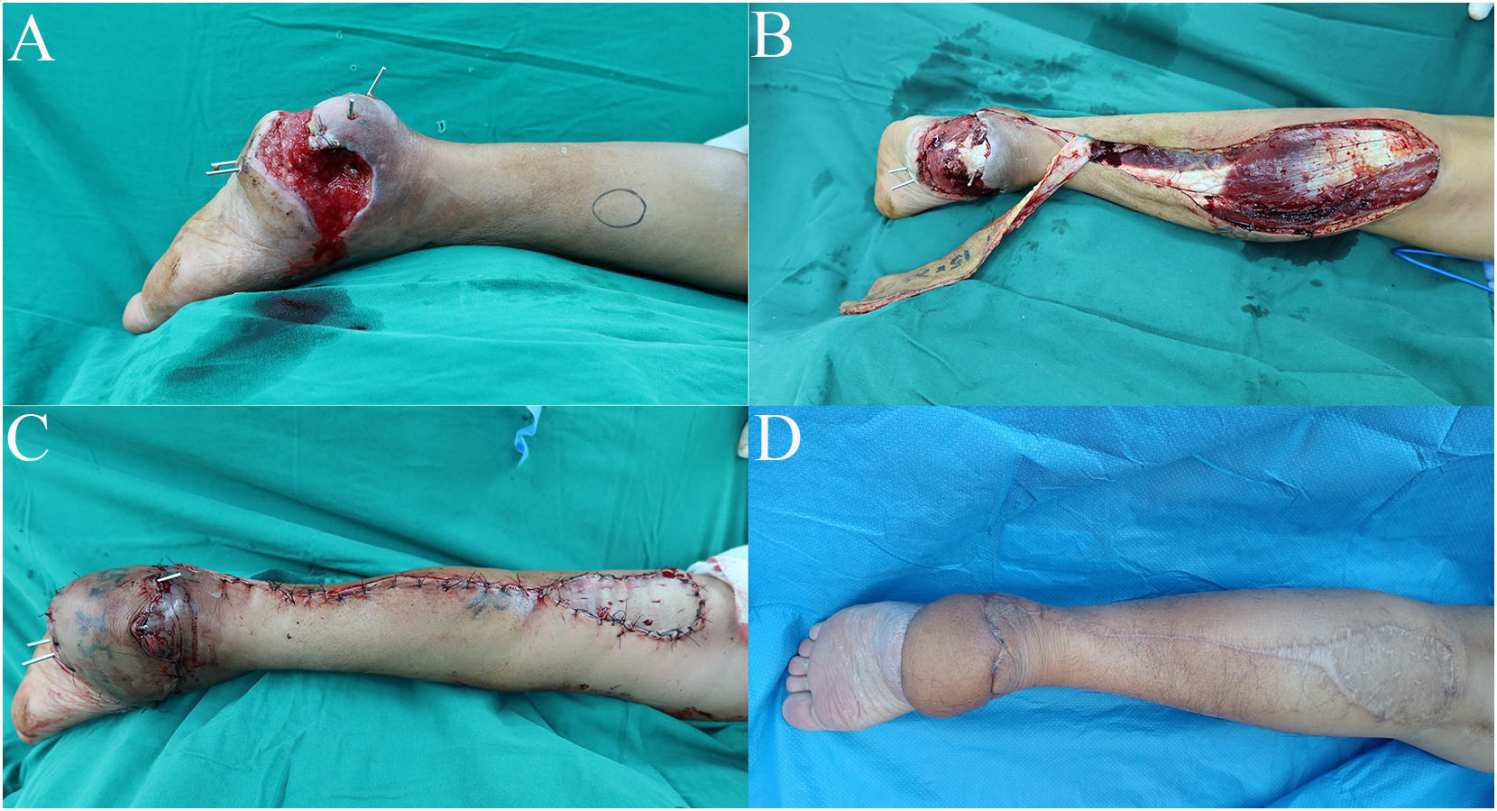
Case 1: Te distally based sural neurocutaneous flaps for reconstruction of the heel soft tissue defect. **(A)** Diabetic wound at the heel. **(B)** Harvest of the distally based sural neurocutaneous flap. **(C)** The defect was reconstructed with a flap, and the donor site was covered with skin graft. **(D)** The flap and the donor site were completely healed at follow-up.

**Figure 2:**
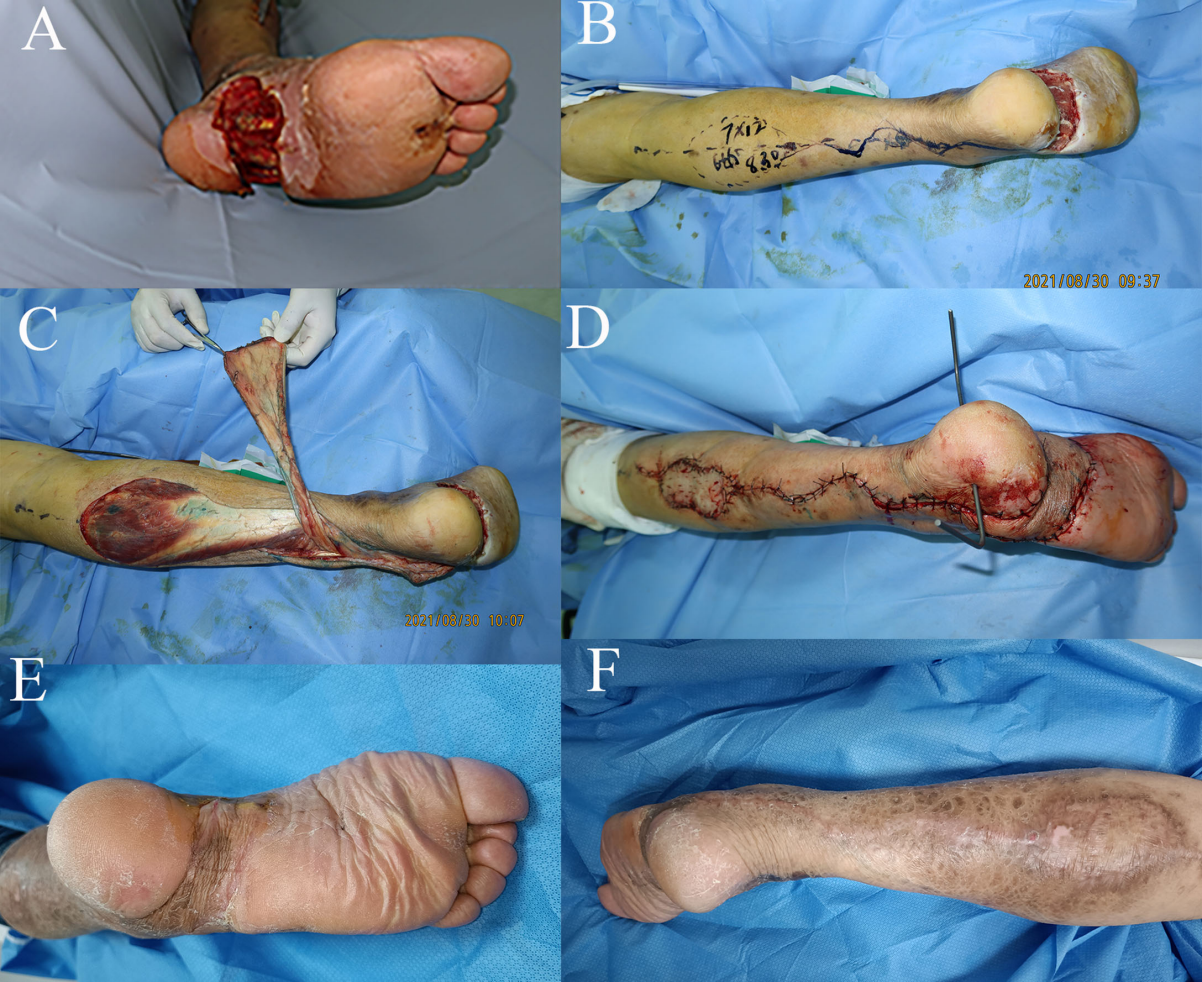
Case 2: The distally based sural neurocutaneous flaps for reconstruction of the sole soft tissue defect. **(A)** Diabetic wound at the sole. **(B)** Design of the flap. **(C)** Harvest of the distally based sural neurocutaneous flap. **(D)** The defect was reconstructed with a flap, and the donor site was covered with skin graft. **(E, F)** The flap and the donor site were completely healed at follow-up.

The size of defect ranged from 5 × 5 to 13 × 6 cm^2^, and the size of flap ranged from 6 × 5 to 15 × 9 cm^2^. The donor site was closed primarily in 10 cases, and split-thickness skin grafts were used in 18 cases and survived entirely. Twenty-two of the 25 flaps survived intact with sufficient blood supply (**Figures 1**, **2**). Two cases had a small superficial necrosis, which was resolved after a change of daily dressing and was healed eventually. In one case, partial necrosis was observed that was managed with minor revision and the use of split-thickness skin graft. No infections or hematomas were encountered in this study. All of 25 flaps gained partial sensorial recovery, except for six patients complaining of anesthesia in the donor site during extended follow-up. No ulcer recurrence was observed in the follow-up. All the patients have good HbA1c levels in the follow-up period. Eighteen of the 25 patients were able to walk unaided, and seven patients with walking stick at follow-up.

## Discussion

As the combination of neuropathy and angiopathy and the propensity to infection, patients with diabetic foot are at greater risk of severe limb ischemia, often with extensive soft tissue loss (12). An amputation is sometimes required when patients suffer from diabetic wound with bone or tendon exposure. It has been found that 40%–70% of all non-traumatic amputations of the lower limbs occur in patients with diabetes (13). Successful reconstruction of distal lower leg wounds in patients with diabetes can, therefore, be limb-saving and lifesaving.

A wide variety of flaps have been reported in the literature for soft tissue defect reconstruction in patients with diabetic foot. Lee et al. (4) conducted a retrospective study of 17 patients with diabetic foot ulcers reconstructed with the proximal lateral leg perforator flap. The authors found one total flap failure and one other flap complicated by venous thrombosis, which was successfully salvaged. Sato et al. (14) reviewed 23 cases of free flap reconstruction for diabetic foot ulcers. Five patients lost their flaps, and the other 16 patients had flap success. Demiri et al. (5) compared outcomes between reverse neurocutaneous and propeller perforator flaps in diabetic foot reconstruction, and uneventful healing was recorded in 20 of the 34 neurocutaneous flaps and in 12 of the 20 propeller flaps. Because of the presence of peripheral neuropathy and microangiopathy in patients with diabetes, the treatment of soft tissue defects in this population is expected to be complicated (15).

Since Masquelet et al. first introduced the concept of neurocutaneous island flaps supplied by the vascular axis of the sensitive superficial nerves in the leg, more anatomical and clinical studies have confirmed the usefulness of these flaps (16, 17). Anatomical basis of the distally based sural flap is represented by vascular axis also accompanied with the sural nerve and the lesser saphenous vein. The blood supply of the sural neurocutaneous flap comes from neurocutaneous perforators from the sural nerve, venocutaneous perforators from the lesser saphenous vein, and septocutaneous perforators from the peroneal artery and the posterior tibial artery. All these perforators connect to each other at the subcutaneous plane, forming a longitudinal chain–linked vascular plexus along sensitive superficial nerves. These characteristics make the distally based sural neurocutaneous flaps predictable and reliable for soft tissue defect coverage of the foot and ankle and less technically demanding than a free flap.

Although the distally based sural neurocutaneous flaps have been widely used for soft tissue coverage in the distal lower extremities, there are few reports on the use of these flaps for diabetic foot treatment. Assi et al. (3) described the sural flap in treating soft tissue defects of the complicated diabetic foot in 14 patients. The authors reported one flap necrosis and three skin edge necrosis, and a hypoesthesia of the lateral aspect of the foot has been noted in 10 patients. Ignatiadis et al. (18) presented their experience with the use of sural fasciocutaneous flaps for the treatment of traumatic wound or diabetic foot in 16 patients. Five cases had a superficial necrosis; two cases experienced a partial skin necrosis, which were treated with a secondary flap; and another case demonstrated a delayed skin healing. In our study, 22 of the 25 flaps survived intact with sufficient blood supply. Two cases had a small superficial necrosis, which was resolved after a change of daily dressing and was healed eventually. In one case, partial necrosis was observed that was managed with minor revision and the use of split-thickness skin graft. Our results were in line with those studies, in which the distally based sural neurocutaneous flaps are useful, reproducible, and reliable in treating soft tissue defects in patients with diabetes with a low frequency of serious complications.

A study by Malokov et al. (19) evaluated the vascular anatomy of the sural flap in patients suffering from arteriopathy. The author demonstrated a theoretical anatomical possibility of using the sural flap in 23 of the 24 amputation specimens with severe vascular disease. Assi et al. (20) conducted a comparative study to analyze the outcomes of the reverse sural flap in reconstructing soft tissue defects in the lower leg and foot, comparing patients with diabetes and trauma. Patients with diabetes were found to have a similar high success rate and a low complication rate when compared with patients with trauma. A study by Kim et al. (21) compared the outcome of propeller perforator flap between diabetic and non-diabetic patients in the distal lower leg reconstruction. The authors found that sex, diabetes, chronic renal failure, and diabetic neuropathy were associated with flap complication and concluded that the propeller perforator flap might not be effective for diabetic foot ulcer reconstruction. Our study demonstrates good outcomes for flap healing in persons with diabetes and foot wounds, in contrast to previous small-scale studies reported in the literature.

In this study, we performed routine preoperative computed tomographic angiography to evaluate the blood supply in the distal lower leg. All of these patients had preservation of at least one major artery. Even in patients with diabetes with compromised circulation, a major vascular axis (most commonly the peroneal artery) remains patent with viable perforators to supply a perforator flap (22). Therefore, this surgical technique did not require a prior revascularization and microsurgery skills.

Therefore, the distally based sural neurocutaneous flap is an optimal choice for reconstruction of foot and ankle defects in patients with diabetic foot. In the appropriate patient, the distally based sural neurocutaneous flap is a simple and effective procedure with reliable results, good thin skin quality, minimal donor site morbidity, and preservation of the leg vessels. A long-term follow-up is required to confirm whether a wide excision with flap reconstruction provides good functional recovery and contributes to maintaining a reasonable quality of life when compared with amputation.

## Data availability statement

The raw data supporting the conclusions of this article will be made available by the authors, without undue reservation.

## Ethics statement

The studies involving human participants were reviewed and approved by the Ethic Review Board of Shanghai Six People’s Hospital affiliated to Shanghai Jiao Tong University. The patients/participants provided their written informed consent to participate in this study. Written informed consent was obtained from the individual(s) for the publication of any potentially identifiable images or data included in this article.

## Author contributions

Conceptualization: HC; investigation: JD; methodology: JD, YZ, and SM; writing—original draft: JD; writing—review and editing: JD, YZ and HC. All authors have read and approved the manuscript and ensure that this is the case.

## Funding

Sponsorship for this study and article processing charges was supported by a grant from the Shanghai Municipal Health Commission (20204Y0430).

## Conflict of interest

The authors declare that the research was conducted in the absence of any commercial or financial relationships that could be construed as a potential conflict of interest.

## Publisher’s note

All claims expressed in this article are solely those of the authors and do not necessarily represent those of their affiliated organizations, or those of the publisher, the editors and the reviewers. Any product that may be evaluated in this article, or claim that may be made by its manufacturer, is not guaranteed or endorsed by the publisher.

## Supplementary material

Supplementary Figure S1 The ulcer was located in the dorsum of foot and covered with the distally based sural neurocutaneous flaps.

Supplementary Figure S2 The ulcer was located in 3-5 toes and lateral dorsum of foot. We have excised 3-5 toes and partial metatarsal bone. The wound was covered with the distally based sural neurocutaneous flaps.
