# Corrigendum: Application of the distally based sural neurocutaneous flaps in the management of foot and ankle defects in patients with diabetic foot

**DOI:** 10.3389/fendo.2022.1054322

**Published:** 2022-11-23

**Authors:** Jiezhi Dai, Yu Zhou, Shasha Mei, Hua Chen

**Affiliations:** ^1^Department of Orthopedic Surgery, Shanghai Jiao Tong University Affiliated Sixth People’s Hospital, Shanghai, China; ^2^Department of Orthopedic Surgery, Civil Aviation Hospital of Shanghai, Shanghai, China; ^3^Department of Anesthesiology, Shanghai Jiao Tong University Affiliated Sixth People’s Hospital, Shanghai, China

**Keywords:** diabetic foot, diabetic wound defect, distally based sural flap, wound healing, foot and ankle reconstruction

In the published article, there was an error in **Figure 1** as published. As the authors were careless in picture editing, they have amended the figure to provide improved clarity. The corrected **Figure 1** and its caption "Case 1: Te distally based sural neurocutaneous flaps for reconstruction of the heel soft tissue defect. **(A)** Diabetic wound at the heel. **(B)** Harvest of the distally based sural neurocutaneous flap. **(C)** The defect was reconstructed with a flap, and the donor site was covered with skin graft. **(D)** The flap and the donor site were completely healed at follow-up" appear below.

**Figure 1:**
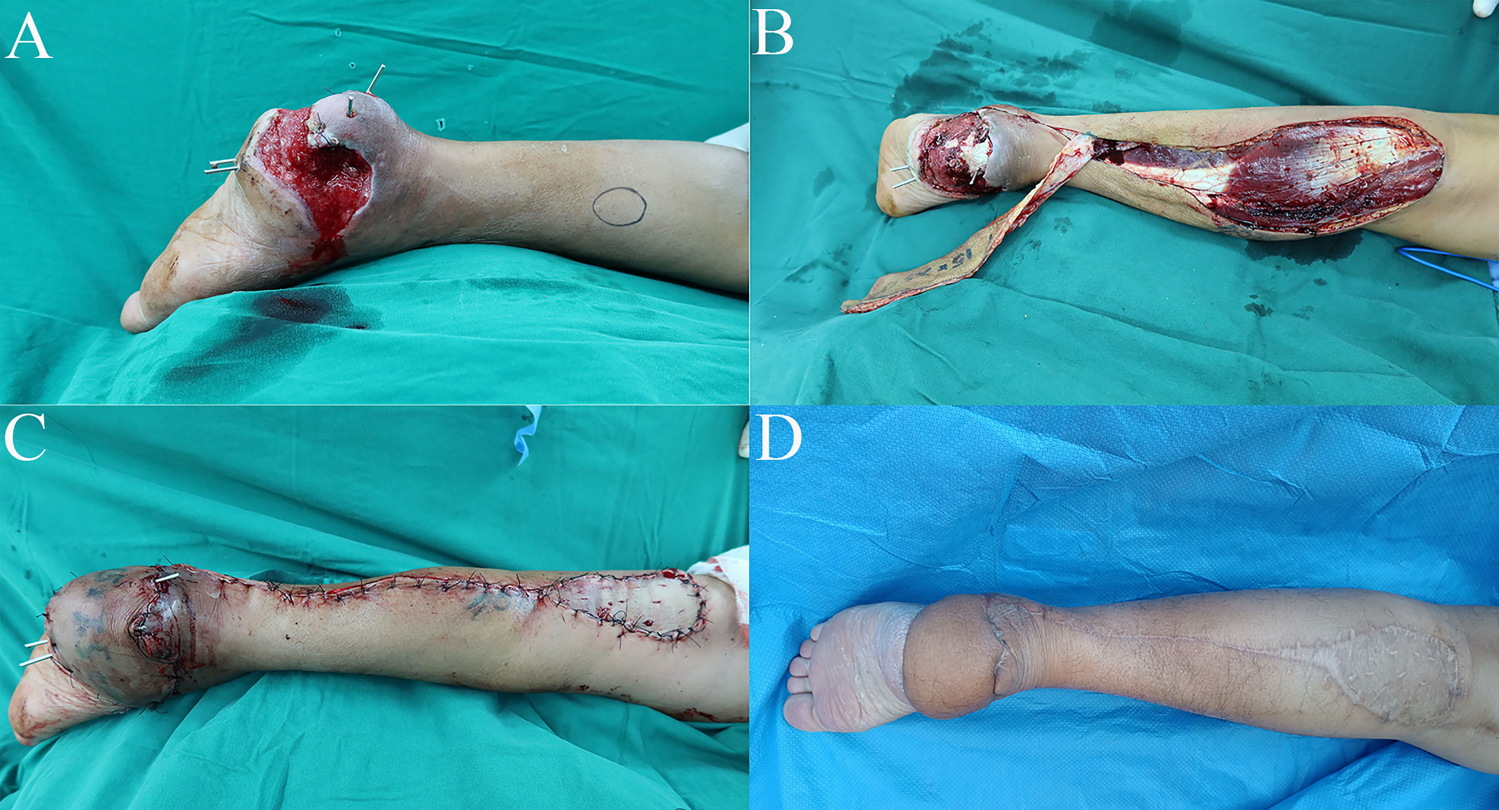
Case 1: Te distally based sural neurocutaneous flaps for reconstruction of the heel soft tissue defect. **(A)** Diabetic wound at the heel. **(B)** Harvest of the distally based sural neurocutaneous flap. **(C)** The defect was reconstructed with a flap, and the donor site was covered with skin graft. **(D)** The flap and the donor site were completely healed at follow-up.

The authors apologize for this error and state that this does not change the scientific conclusions of the article in any way. The original article has been updated.

## Publisher’s note

All claims expressed in this article are solely those of the authors and do not necessarily represent those of their affiliated organizations, or those of the publisher, the editors and the reviewers. Any product that may be evaluated in this article, or claim that may be made by its manufacturer, is not guaranteed or endorsed by the publisher.

