# Supplementary figures and images for: Application of the distally based sural neurocutaneous flaps in the management of foot and ankle defects in patients with diabetic foot
Source: Front Endocrinol (Lausanne). 2022 Sep 23;13:1009714. doi: 10.3389/fendo.2022.1009714 (PMC9537483; doi:10.3389/fendo.2022.1009714)

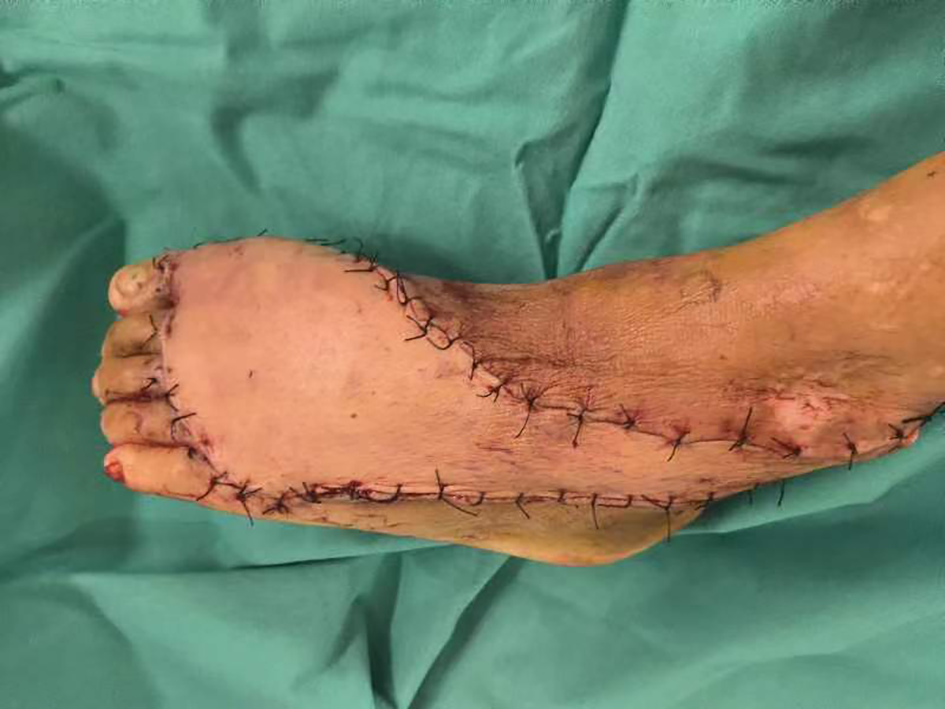

Supplement: Supplementary Figure S1 — The ulcer was located in the dorsum of foot and covered with the distally based sural neurocutaneous flaps. [file Image_1.tif]

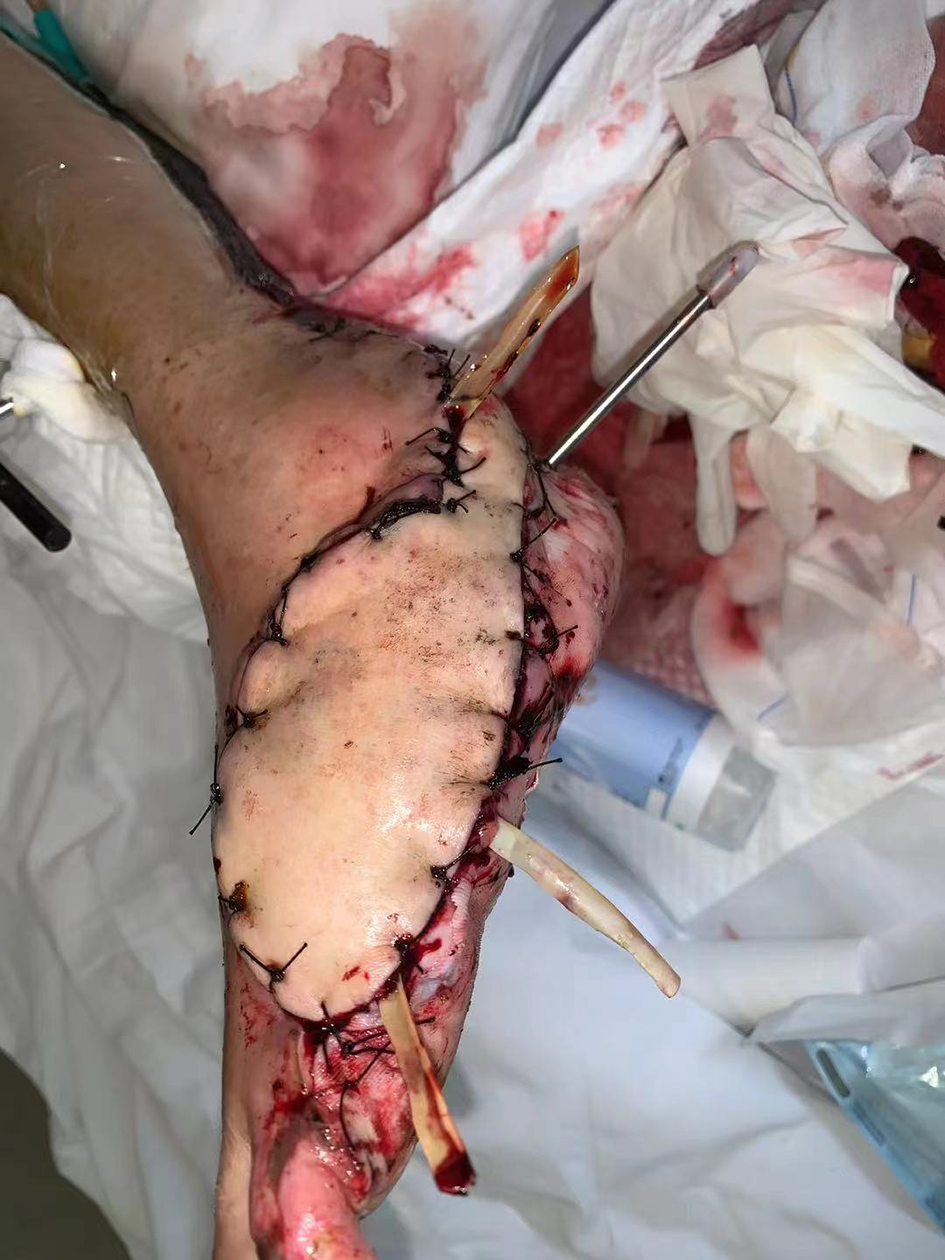

Supplement: Supplementary Figure S2 — The ulcer was located in 3-5 toes and lateral dorsum of foot. We have excised 3-5 toes and partial metatarsal bone. The wound was covered with the distally based sural neurocutaneous flaps. [file Image_2.tif]
